# Whom to treat? Factors associated with chemotherapy recommendations and outcomes among patients with NHL at the Uganda Cancer Institute

**DOI:** 10.1371/journal.pone.0191967

**Published:** 2018-02-01

**Authors:** Manoj Menon, Anna Coghill, Innocent Mutyaba, Fred Okuku, Warren Phipps, John Harlan, Jackson Orem, Corey Casper

**Affiliations:** 1 Vaccine and Infectious Disease Division, Fred Hutchinson Cancer Research Center, Seattle, WA, United States of America; 2 Department of Medicine, University of Washington, Seattle, WA, United States of America; 3 Uganda Cancer Institute, Kampala, Uganda; 4 Hutchinson Centre Research Institute, Kampala, Uganda; 5 Infectious Disease Research Institute, Seattle, WA, United States of America; European Institute of Oncology, ITALY

## Abstract

**Introduction:**

Cancer treatment options in sub-Saharan Africa are scarce despite an increasing burden of disease. Identification of those cancer patients who would benefit most from the limited resources available would allow broader and more effective therapy.

**Methods:**

We conducted a retrospective analysis of patients over the age of 18 at the time of a pathologic diagnosis of NHL between 2003 and 2010 who were residents of Kyandondo County (Uganda) and presented to the Uganda Cancer Institute for care.

**Results:**

A total of 128 patients were included in this analysis. Chemotherapy was recommended to 117 (91.4%) of the patients; the odds of recommending chemotherapy decreased for each additional month of reported symptoms prior to diagnosis.

Of the 117 patients to whom chemotherapy was recommended, 111 (86.7%) patients received at least 1 cycle of chemotherapy; HIV infected patients, as well as those with a lower hemoglobin and advanced disease at the time of diagnosis were significantly less likely to complete therapy. Among the patients who initiated chemotherapy, twenty patients died prior to treatment completion (including nine who died within 30 days). Hemoglobin level at the time of presentation was the only variable associated with early mortality in the adjusted model.

**Conclusion:**

In resource-poor areas, it is essential to align health care expenditures with interventions likely to provide benefit to affected populations. Targeting cancer therapy to those with a favorable chance of responding will not only save limited resources, but will also prevent harm in those patients unlikely to realize an effect of cancer-directed therapy.

## Introduction

As mortality attributable to communicable diseases continues to fall in resource-limited settings, cancer has become a large and growing threat to the health of populations in these regions. Current estimates indicate that cancer is now responsible for more deaths globally than HIV, malaria, and tuberculosis combined and future estimates project an even greater relative burden.[1, 2]Due in part to population growth, aging, and lifestyle factors (e.g. tobacco use) the cumulative burden and mortality rates attributed to cancer are expected to increase in the next 20 years globally.[3] Resource-limited regions, including sub-Saharan Africa (SSA), will bear the brunt of this increase. Nearly half a million people are estimated to die of cancer in 2020 in SSA, and the incidence of cancer will increase at least 40% between now and then.[1]

Unfortunately, cancer treatment options in much of SSA are often hampered by a weak or non-existent oncology health care infrastructure, resulting in advanced disease at presentation and the limited availability of effective but cost prohibitive chemotherapy. Consequently, patient outcomes are typically poor. In a retrospective analysis of over 150 patients with newly diagnosed non-Hodgkin’s lymphoma (NHL) in Uganda, researchers found that nearly 40 percent of patients died within in the first 12 months and that among those who died within the first year, the median overall survival was only 2 months. Untreated HIV infection, low hemoglobin, and constitutional symptoms independently predicted a worse outcome.[4] Similar findings have been demonstrated among pediatric patients in Uganda as well, as the presence of constitutional symptoms, low hemoglobin level, and poor performance status were predictive of 30 day mortality.[5] These same factors have been prospectively shown to be associated with poor prognosis and early mortality in resource-abundant regions as well.[6–8]

Given the late-stage of presentation typical in resource-poor regions, it is also less clear whether early mortality represents disease progression or treatment related toxicity. Consequently, in resource poor settings there is an unmet need regarding prognostic factors that would identify patients likely to benefit from standard cancer directed therapy and those patients who would be better served by alternative therapeutic options, including palliative and supportive measures. Such data may inform clinical decision-making and help optimize the limited resources available.

Here we describe characteristics of patients with a new diagnosis of pathologically confirmed NHL presenting for care at Uganda Cancer Institute, identify patient factors associated with the recommendation to receive and complete cancer-directed therapy, and document factors associated with early mortality among patients receiving chemotherapy.

## Methods

We conducted an analysis of patients age 18 or older at the time of diagnosis of NHL between 2003 and 2010 who were residents of Kyadondo County (Uganda) and part of a retrospective cohort study previously described.[9] Kyadondo County comprises Kampala city, the capital of Uganda, and its peri-urban areas. Cases were identified from the Kampala Cancer Registry (KCR)–a population-based cancer registry covering Kyadondo County and provides among the longest series of cancer incidence in SSA. We transferred patient lists from KCR were to the Uganda Cancer Institute (UCI), the nation’s sole cancer center, and Mulago Hospital, a university teaching hospital, which are located in Kampala City. Given that it is not mandatory to report cases of cancer to the registry, we identified eligible patients from patient records at the UCI and Mulago Hospital who had not yet been recorded in the KCR. Pathological evaluation was performed locally and all patients received care at the UCI. The UCI is an 80-bed public medical care facility and cares for approximately 200 patients daily in both the inpatient and outpatient setting. Medications on the formulary used in the treatment of NHL (e.g. cyclophosphamide, doxorubicin, vincristine, prednisone [CHOP]) are provided free of charge. Ancillary medications that are not on the formulary, including rituximab and growth factor support can be purchased privately and administered at the UCI. However, given the expense of these medications the use of such ancillary medications remains rare.

We reviewed medical records were reviewed for all eligible patients. Patients without a pathologically confirmed diagnosis were not included in this analysis. Additionally, we excluded patients with Hodgkin Lymphoma, lymphoma not otherwise specified, or those determined to have a relapsed malignancy from this analysis.

Demographic (e.g. sex, age at diagnosis), clinical (e.g. symptom duration, comorbidities, physical exam findings, clinical stage) and laboratory data (e.g. hemoglobin, LDH, albumin) at the time of diagnosis were abstracted from the medical record. When available, we abstracted radiographic data, typically an abdominal ultrasound and/or chest radiograph. As per standard clinical staging systems, early stage disease included either the involvement of a single lymph node region (Stage 1) or the presence of 2 or more lymph node regions on the same side of the diaphragm (Stage 2). We characterized advanced disease by involvement of lymph node regions on both sides of the diaphragm (Stage 3) or diffuse disease (Stage 4).[10] In the event that clinical stage was not recorded in the medical record, a study physician at UCI reviewed the medical record and assigned a clinical stage. HIV status was ascertained by either the presence of results of HIV antibody testing, documentation of care at a local HIV treatment facility, or documentation of HIV status in the clinical notes. We assessed medical records for the presence of the following symptoms or signs: palpable mass, dysphagia, bone pain, edema, fever, hepatomegaly, bleeding, fatigue, ascites, wasting, anemia, and pleural effusion. A symptom score, a composite measure of the individual symptoms reported, was created; each symptom was given the same weight. Similarly, a comorbidity index was calculated with each coexisting medical illness contributing equally to this measurement. Performance status data were not routinely recorded in the medical record and were therefore not included in this analysis.

The primary outcome measure was whether chemotherapy was recommended by the treating clinician. We assessed whether demographic, clinical, and laboratory measurements were associated with the recommendation for treatment with chemotherapy using logistic regression models. Variables with a p value < 0.20 in the unadjusted model were included in the adjusted logistic regression analysis.[11] We conducted a similar analysis among those patients who completed the recommended course of chemotherapy.

In order to assess early mortality, we calculated 30-day survival for patients as well as evaluated those who died prior to completion of the first course of chemotherapy. Survival time was calculated by measuring the time difference between the date of either the initial presentation or initial pathologic diagnosis, whichever occurred earlier, and date of death. Patients with missing survival data were excluded from the early mortality analyses. Bivariate associations between demographic, clinical, and laboratory features and early mortality were analyzed using logistic regressions. Features which were associated with early mortality on bivariate analyses (p < .20) were included in a multivariate analysis.

This study was approved by the Makerere University College of Health Sciences Research Ethics Committee (Kampala, Uganda) and the Fred Hutchinson Cancer Research Center’s Institutional Review Board (Seattle, WA, USA). All data were fully anonymized prior to access and analysis.

## Results

A total of 128 patients presenting to the Uganda Cancer Institute (UCI) between 2003 and 2010 with a pathologic diagnosis of NHL were included in this analysis. The median age at presentation for care was 39.5 years (range 19–82 years); 46% (59/128) of the patients were female. Over half of the patients (57.0%; 73/128) were HIV positive; other comorbidities such as tuberculosis (15.6%; 19/128), cardiovascular disease (6.7%; 8/128) and diabetes (5.7%; 7/128) were less common. The median baseline hemoglobin level was 10.8 g/dl (IQR 8.5–12.6) and the median LDH was elevated at 416 units/L. (Table 1).

**Table 1:** Demographic, clinical, and lab characteristics (N = 128).

Characteristic	n (%)
Gender	
Male	69 (53.9)
Female	59 (46.1)
Age, in years, median (range)	39.5 (19–82)
Presenting symptoms	
Mass	98 (76.6)
Fever	73 (57.0)
Wasting	67 (52.3)
Anemia	48 (37.5)
Symptom duration prior to presentation, in months, median (range)	4 (1–96)
Symptom score, median (range)	3 (0–9)
Stage at presentation	
1	4 (3.3)
2	11 (9.2)
3	43 (35.8)
4	62 (51.7)
HIV—infected	73 (57.0)
Comorbid conditions	
Tuberculosis	19 (15.6)
Lung disease	9 (7.4)
Liver disease	9 (7.4)
Cardiovascular disease	8 (6.7)
Diabetes	7 (5.7)
Laboratory results at presentation	
Hemoglobin (g/dL), median (IQR)	10.8 (8.6–12.6)
LDH (units/L), median	416
Albumin (g/L), median	33
Chemotherapy recommended	117 (91.4)
Chemotherapy received (at least 1 cycle)	111 (86.7)
Completed course of therapy	58 (48.3)

Nearly 90% (105/128) of the patients presented with advanced disease. The vast majority of patients (97.0%; 124/128) reported at least 1 symptom at presentation and a median of 3 separate symptoms. Fever (57.0%; 73/128), a palpable mass (76.6%; 98/128), and wasting (52.3%; 67/128) were the most common presenting symptoms. Chemotherapy was recommended to 117 (91.4%) of the patients and was initiated on 111 of those patients. Of those patients who started chemotherapy, 58 (48.3%) patients completed the recommended course of chemotherapy (nearly all of whom received 6 cycles of chemotherapy). Of the 53 patients who did not complete the complete course of chemotherapy, 18 (34%) died prior to the completion of therapy; the remainder were either lost to follow-up (25; 47%) or the specific reason was not known (10; 18.9%). Notably, financial constraints did not preclude completion of chemotherapy for any of the patients.

On bivariate analysis, increasing age, shorter symptom duration, and lower stage at presentation were associated with an increased odds of receiving a recommendation for chemotherapy. In the adjusted model, only symptom duration (OR = .95; 95% CI [.92,.99]) remained significantly associated with a decreased odds of recommending chemotherapy by 5% for each additional month of symptom duration at presentation. (Table 2). Of the 11 patients who were not recommended to receive chemotherapy, 6 (54.5%) died within 30 days of presentation.

**Table 2:** Factors associated with recommendation for chemotherapy.

	Bivariate		Multivariate	
	Odds Ratio	95% CI	Odds Ratio	95% CI
Female	2.44	[.62,9.69]	NA	
Age (each additional year)	**1.04**	**[.98,1.09]**	1.04	[.98, 1.11]
Symptom score (each additional symptom)	0.91	[.64,1.29]	NA	
Symptom duration (each additional month)	**0.97**	**[.93,1.00]**	**0.95**	**[.92, .99]**
HIV-infected	0.74	[.21,2.66]	NA	
Comorbidity score	+			
Hemoglobin (each unit increase g/dL)	1.07	[.87, 1.31]	NA	
Stage at presentation*	**0.23**	**[.06, 1.05]**	0.19	[.04, 1.02]

* All patients with limited stage disease (i.e. Stage 1 or 2) were recommended to receive chemotherapy.

**+ All patients with a comorbidity score** **≥** **1 were recommended to receive chemotherapy**

On bivariate analysis, HIV-uninfected patients, as well as those patients presenting with a longer duration of symptoms, higher hemoglobin, and less advanced stage of disease were more likely to complete the course of chemotherapy. In the adjusted model, patients with a higher hemoglobin (OR = 1.28; 95% CI [1.06,1.53]) and those with an earlier stage of disease (OR = 0.34; 95% CI [0.18, 0.67]) remained more likely to complete therapy than their counterparts (Table 3).

**Table 3:** Factors associated with completion of first course of therapy.

	Bivariate		Multivariate	
	Odds Ratio	95% CI	Odds Ratio	95% CI
Female	0.76	[.37, 1.56]		
Age (each additional year)	1.01	[.98, 1.03]		
Symptom score (each additional symptom)	0.91	[.73 1.13]		
Symptom duration (each additional month)	1.05	[.99, 1.11]	1.06	[.99, 1.14]
HIV infection	0.51	[.25, 1.07]	.40	[.16, 1.05]
Comorbidity index	0.86	[.56, 1.34]		
Hemoglobin (each unit increase) g/dL)	1.31	[1.13, 1.51]	**1.28**	**[1.06, 1.53]**
Stage at presentation (each additional stage)	0.29	[.16, .54]	**0.34**	**[.18, .67]**

Of the 111 patients who started chemotherapy, nine patients (8.1%) died within 30 days of diagnosis. On the bivariate analysis, patients with a lower hemoglobin and advanced stage of disease at presentation were more likely to die within 30 days. In adjusted analyses, only hemoglobin at time of presentation (OR = .75; 95 CI [.59, .97] remained associated with early mortality (Table 4).

**Table 4:** Factors associated with early mortality.

	Bivariate		Multivariate	
	Odds Ratio	95% CI	Odds Ratio	95% CI
Female	0.93	[.24, 3.64]	NA	
Age (each additional year)	1.01	[.96, 1.06]	NA	
Symptom score (each additional symptom)	0.80	[.49, 1.30]	NA	
Symptom duration (each additional month)	0.99	[.92, 1.07]	NA	
HIV-infected	0.58	[.15, 2.27]	NA	
Comorbidity score (each additional comorbidity)	0.45	[.13, 1.53]	NA	
Hemoglobin (each unit increase) g/dL)	**0.75**	**[.59, .94]**	**0.75**	**[59, .97]**
Stage at presentation (each additional stage)*	**3.22**	**[.76, 13.7]**	2.4	[.58, 10.3]

*only patients with advanced stage of disease at presentation died within 30 days of treatment

Among patients who received at least 1 cycle of chemotherapy and who were lost to follow-up (n = 42; 37.8%), the mean age was 42.6 and comparable to those patients with complete follow-up. Patients lost to follow up had comparable levels of hemoglobin and presented at a similar stage of disease as their counterparts. However, patients lost to follow-up were more likely to be HIV positive (71.4% vs. 46.4%; p < .05).

## Discussion

In our study, the vast majority of patients were symptomatic and presented with an advanced stage of disease; the recommendation for treatment was nearly universal. This recommendation is similar in resource-abundant areas where therapeutic decision-making among patients with NHL is typically independent of the presence of validated poor prognostic factors, including poor performance status, anemia, and age, given the ability to cure a subset of patients with treatment and the dire prognosis in the absence of treatment. Indeed, most patients with NHL receive the same dose intense regimen independent of age and performance status.[12–16]

Although the sample size is small, patients with a longer duration of symptoms were less likely to receive a recommendation for chemotherapy. Although we do not know the clinical decision-making involved, the failure to recommend therapy in these patients with prolonged symptoms may have been due to perceived inability to tolerate the combination chemotherapy. Multiple studies have documented the prognostic utility of a patient’s performance status in determining the utility of and patient’s tolerance of chemotherapy, however the data are primarily limited to solid malignancies.[17, 18] Similarly, a recent systematic review of over 200 studies found that shorter time period between symptom onset and diagnosis was associated with an improved outcome[19].

If a patient were to receive any chemotherapy, patients with advanced stage of disease, and lower hemoglobin were less likely to complete therapy. Given the consequences of anemia, a low hemoglobin may serve as a proxy for functional status, which has been validated as both a predictor of response to therapy and in prognosis of outcome in both resource-poor and resource-abundant setting. [4, 6–8, 17, 18] A low hemoglobin level may also have limited the patient’s ability to tolerate potentially curative cancer-directed therapy. Alternatively, anemia may be involved in the causal pathway in the pathogenesis of NHL, such that bone marrow involvement (and the consequent decreased hemoglobin) is indicative of more aggressive disease. Similarly, both advanced stage and low hemoglobin were related to early death among those treated with chemotherapy on the bivariate analysis. Although the direction of the relationship can not be determined from this analysis, this relationship was only maintained for those with a low hemoglobin in the adjusted analysis.

Given their independent effect on response to therapy and overall survival, clinical prognostic indices are often used in resource-abundant countries to identify which patients will derive a benefit from cancer-directed therapy and which patients may be better served by supportive measures. The International Non-Hodgkin’s Lymphoma Prognostic Factors Project developed a model based on clinical factors to predict outcome among patients with newly diagnosed NHL in the pre-rituximab era.[20] These researchers found that five variables (i.e. age, stage of disease, LDH, performance status, and extent of extranodal disease involvement) could risk stratify patients with aggressive NHL into four prognostic groups and serve as the International Prognostic Index (IPI). Although the prognostic utility of such variables is less well validated in the era of targeted therapy, including rituximab, older data are particularly relevant in sub-Saharan Africa where the use of rituximab and other targeted therapies are not readily available. Indeed, the treatment for the majority of NHL patients with CHOP (without the use of rituximab or other targeted therapy) remains the same now in Uganda as it was during the period of this analysis. Notably, the IPI did not include hemoglobin in its prognostic index. However, a hemoglobin less than 12 g/dL (in addition to age, stage, number of nodal areas, and elevated LDH) was prognostic among patients with follicular lymphoma as per the Follicular Lymphoma International Prognostic Index (FLIPI).[21]

Recently, colleagues documented the lack of survival benefit and the detrimental effect of quality of life among patients with end-stage cancer (i.e. a physician-estimated life expectancy of less than or equal to 6 months) and a good performance status receiving chemotherapy[22]. The toxicity of combination chemotherapy is likely even more profound in resource-limited settings given the lack of supportive therapies. A retrospective study of elderly patients with either intermediate or high grade NHL treated with combination chemotherapy (i.e. CHOP) in Peru found that there were 35 deaths among the 267 patients treated (13.1%); the majority of these treatment related deaths were secondary to infection.[23] A comprehensive and systematic review of 17 randomized controlled trials revealed that the use of prophylactic granulocyte-colony stimulating factor (G-CSF) decreased the risk of febrile neutropenia and early death–including infection related mortality. [24]Indeed, current guidance from the American Society of Clinical Oncology, the European Organization for Research and Treatment of Cancer, the Infectious Disease Society of America, and the National Comprehensive Cancer Network recommend primary prophylaxis when the incidence of febrile neutropenia is expected to be greater than 20%.[25–28] In Uganda, the use of G-CSF to treat or prevent chemotherapy-induced neutropenia is not routine and generally unaffordable. As such, the risk of potentially fatal infections is high.

In the setting of cancer-directed therapy in resource-abundant areas, early mortality is often related to sequelae from the treatment rather than progression of disease and is termed treatment-related mortality, however its definition of has not been well harmonized.[29] Regardless, given the potential for cure, the vast majority of patients with NHL in resource-abundant areas are treated independent of prognosis and risk of treatment related morbidity or mortality. However, the toxicity of cancer-directed therapy, including myelosuppression, is likely more severe and lethal in resource-poor regions where the risk of infection is high and the availability of supportive therapies, including blood transfusion and prophylactic antibiotics, is limited.[30, 31] Although widely used, the IPI has not been prospectively validated in resource-poor regions of the world. Previous retrospective data in Uganda have identified predictors of poor survival, including low hemoglobin and presence of constitutional symptoms, among patients older than age 13 with NHL.[4] In our study, only a low hemoglobin was associated with early mortality. Regardless, locally relevant and validated prognostic indices are necessary.

The primary limitation of this study is the large percentage of patients who were lost to follow up, a challenge in resource-poor settings with a limited health care infrastructure. While data are relatively complete with regard to treatment recommendations, data are incomplete regarding outcome. As such, we are unable to evaluate treatment efficacy. Patients with incomplete outcome data were excluded from the early mortality analysis but patients who were lost to follow-up were more likely to HIV positive. Additionally, although patients were diagnosed based on pathologic evaluation, recent data suggest concerns with both false positive and false negative results. [32]. Finally, the histologic subtype of NHL includes both indolent and aggressive variants. Nevertheless, we feel that our findings represent the reality of clinical care in resource-poor settings but highlight the need for complete prospective data on patients with newly diagnosed NHL in SSA. Such information will allow clinicians to develop and validate a locally-relevant prognostic index and determine whether such measures inform survival and optimize treatment decisions. Although relevant globally, the allocation of scarce health care resources, including cytotoxic chemotherapy, to those patients that will be most likely to derive a meaningful benefit is imperative in resource-poor regions. Targeting therapy will not only save limited resources, it will also prevent harm in those patients unlikely to realize an effect of cancer-directed therapy.

## References

[1] Ferlay J, Soeriomataram I, Ervik M, Dikshit R, Eser S, Mathers C, et al, http://globocan.iarc.fr. Af. Cancer Incidence and Mortality Worldwide Lyon, France: International Agency for Research on Cancer.; 2013. IARC CancerBase No. 11 [Internet].:; GLOBOCAN 2012 v1.0,:[GLOBOCAN 2012 v1.0,].

[2] Garcia M JA, Ward EM, Center MM, Hao Y, Siegel RL, Thun MJ. Global Cancer Facts and Figures. American Cancer Society. 2007.

[3] Bray F, Jemal A, Grey N, Ferlay J, Forman D. Global cancer transitions according to the Human Development Index (2008–2030): a population-based study. The lancet oncology. 2012;13(8):790–801. Epub 2012/06/05. doi: 10.1016/S1470-2045(12)70211-5 . 22658655 10.1016/S1470-2045(12)70211-5

[4] Bateganya MH, Stanaway J, Brentlinger PE, Magaret AS, Wald A, Orem J, et al Predictors of survival after a diagnosis of non-Hodgkin lymphoma in a resource-limited setting: a retrospective study on the impact of HIV infection and its treatment. Journal of acquired immune deficiency syndromes (1999). 2011;56(4):312–9. Epub 2011/02/26. doi: 10.1097/QAI.0b013e31820c011a ; PubMed Central PMCID: PMCPMC3065203. 21350364 10.1097/QAI.0b013e31820c011a PMC3065203

[5] Mutyaba IOJ, Wabinga H, Phipps W, Casper C. Access to cancer chemotherapy and predictors of early mortality for childhood cancers in Uganda In: J Clin Oncol 31 sa, editor. ASCO; Chicago 2013.

[6] Hauser CA, Stockler MR, Tattersall MH. Prognostic factors in patients with recently diagnosed incurable cancer: a systematic review. Supportive care in cancer: official journal of the Multinational Association of Supportive Care in Cancer. 2006;14(10):999–1011. doi: 10.1007/s00520-006-0079-9 . 16708213 10.1007/s00520-006-0079-9

[7] Maltoni M, Pirovano M, Scarpi E, Marinari M, Indelli M, Arnoldi E, et al Prediction of survival of patients terminally ill with cancer. Results of an Italian prospective multicentric study. Cancer. 1995;75(10):2613–22. . 7537625 10.1002/1097-0142(19950515)75:10<2613::aid-cncr2820751032>3.0.co;2-1

[8] Reuben DB, Mor V, Hiris J. Clinical symptoms and length of survival in patients with terminal cancer. Archives of internal medicine. 1988;148(7):1586–91. . 3382303

[9] Coghill AE, Newcomb PA, Madeleine MM, Richardson BA, Mutyaba I, Okuku F, et al Contribution of HIV infection to mortality among cancer patients in Uganda. Aids. 2013 doi: 10.1097/01.aids.0000433236.55937 . 23921614 10.1097/01.aids.0000433236.55937.cb PMC4319357

[10] Lister TA, Crowther D, Sutcliffe SB, Glatstein E, Canellos GP, Young RC, et al Report of a committee convened to discuss the evaluation and staging of patients with Hodgkin's disease: Cotswolds meeting. Journal of clinical oncology: official journal of the American Society of Clinical Oncology. 1989;7(11):1630–6. Epub 1989/11/01. doi: 10.1200/JCO.1989.7.11.1630 . 2809679 10.1200/JCO.1989.7.11.1630

[11] Bursac Z, Gauss CH, Williams DK, Hosmer DW. Purposeful selection of variables in logistic regression. Source code for biology and medicine. 2008;3:17 Epub 2008/12/18. doi: 10.1186/1751-0473-3-17 ; PubMed Central PMCID: PMCPMC2633005. 19087314 10.1186/1751-0473-3-17 PMC2633005

[12] Bairey O, Benjamini O, Blickstein D, Elis A, Ruchlemer R. Non-Hodgkin's lymphoma in patients 80 years of age or older. Annals of oncology: official journal of the European Society for Medical Oncology / ESMO. 2006;17(6):928–34. Epub 2006/03/02. doi: 10.1093/annonc/mdl034 . 16507563 10.1093/annonc/mdl034

[13] Coiffier B, Lepage E, Briere J, Herbrecht R, Tilly H, Bouabdallah R, et al CHOP chemotherapy plus rituximab compared with CHOP alone in elderly patients with diffuse large-B-cell lymphoma. The New England journal of medicine. 2002;346(4):235–42. Epub 2002/01/25. doi: 10.1056/NEJMoa011795 . 11807147 10.1056/NEJMoa011795

[14] Cuttner J, Wallenstein S, Troy K. Non-Hodgkin's lymphoma in patients 70 years of age or older: factors associated with survival. Leukemia research. 2002;26(5):447–50. Epub 2002/03/28. . 11916517 10.1016/s0145-2126(01)00163-1

[15] Gregory SA, Trumper L. Chemotherapy dose intensity in non-Hodgkin's lymphoma: is dose intensity an emerging paradigm for better outcomes? Annals of oncology: official journal of the European Society for Medical Oncology / ESMO. 2005;16(9):1413–24. Epub 2005/06/04. doi: 10.1093/annonc/mdi264 . 15932900 10.1093/annonc/mdi264

[16] Pfreundschuh M, Trumper L, Kloess M, Schmits R, Feller AC, Rube C, et al Two-weekly or 3-weekly CHOP chemotherapy with or without etoposide for the treatment of elderly patients with aggressive lymphomas: results of the NHL-B2 trial of the DSHNHL. Blood. 2004;104(3):634–41. Epub 2004/03/16. doi: 10.1182/blood-2003-06-2095 . 15016643 10.1182/blood-2003-06-2095

[17] Oken MM, Creech RH, Tormey DC, Horton J, Davis TE, McFadden ET, et al Toxicity and response criteria of the Eastern Cooperative Oncology Group. American journal of clinical oncology. 1982;5(6):649–55. . 7165009

[18] Buccheri G, Ferrigno D, Tamburini M. Karnofsky and ECOG performance status scoring in lung cancer: a prospective, longitudinal study of 536 patients from a single institution. European journal of cancer (Oxford, England: 1990). 1996;32A(7):1135–41. . 8758243 10.1016/0959-8049(95)00664-8

[19] Neal RD, Tharmanathan P, France B, Din NU, Cotton S, Fallon-Ferguson J, et al Is increased time to diagnosis and treatment in symptomatic cancer associated with poorer outcomes? Systematic review. British journal of cancer. 2015;112 Suppl 1:S92–107. Epub 2015/03/04. doi: 10.1038/bjc.2015.48 ; PubMed Central PMCID: PMCPmc4385982. 25734382 10.1038/bjc.2015.48 PMC4385982

[20] Project TIN-HsLPF. A Predictive Model for Aggressive Non-Hodgkin's Lymphoma. New England Journal of Medicine. 1993;329(14):987–94. doi: 10.1056/NEJM199309303291402 . 8141877 10.1056/NEJM199309303291402

[21] Solal-Celigny P, Roy P, Colombat P, White J, Armitage JO, Arranz-Saez R, et al Follicular lymphoma international prognostic index. Blood. 2004;104(5):1258–65. Epub 2004/05/06. doi: 10.1182/blood-2003-12-4434 . 15126323 10.1182/blood-2003-12-4434

[22] Prigerson HG, Bao Y, Shah MA, Paulk ME, LeBlanc TW, Schneider BJ, et al Chemotherapy Use, Performance Status, and Quality of Life at the End of Life. JAMA oncology. 2015 Epub 2015/07/24. doi: 10.1001/jamaoncol.2015.2378 . 26203912 10.1001/jamaoncol.2015.2378 PMC4828728

[23] Gomez H, Hidalgo M, Casanova L, Colomer R, Pen DL, Otero J, et al Risk factors for treatment-related death in elderly patients with aggressive non-Hodgkin's lymphoma: results of a multivariate analysis. Journal of clinical oncology: official journal of the American Society of Clinical Oncology. 1998;16(6):2065–9. Epub 1998/06/17. doi: 10.1200/JCO.1998.16.6.2065 . 9626205 10.1200/JCO.1998.16.6.2065

[24] Kuderer NM, Dale DC, Crawford J, Lyman GH. Impact of Primary Prophylaxis With Granulocyte Colony-Stimulating Factor on Febrile Neutropenia and Mortality in Adult Cancer Patients Receiving Chemotherapy: A Systematic Review. Journal of Clinical Oncology. 2007;25(21):3158–67. doi: 10.1200/JCO.2006.08.8823 17634496 10.1200/JCO.2006.08.8823

[25] Aapro MS, Bohlius J, Cameron DA, Dal Lago L, Donnelly JP, Kearney N, et al 2010 update of EORTC guidelines for the use of granulocyte-colony stimulating factor to reduce the incidence of chemotherapy-induced febrile neutropenia in adult patients with lymphoproliferative disorders and solid tumours. European journal of cancer (Oxford, England: 1990). 2011;47(1):8–32. Epub 2010/11/26. doi: 10.1016/j.ejca.2010.10.013 . 21095116 10.1016/j.ejca.2010.10.013

[26] Freifeld AG, Bow EJ, Sepkowitz KA, Boeckh MJ, Ito JI, Mullen CA, et al Clinical practice guideline for the use of antimicrobial agents in neutropenic patients with cancer: 2010 update by the infectious diseases society of america. Clinical infectious diseases: an official publication of the Infectious Diseases Society of America. 2011;52(4):e56–93. Epub 2011/01/25. doi: 10.1093/cid/cir073 . 21258094 10.1093/cid/cir073

[27] NCCN. National Comprehensive Cancer Network. Myeloid growth factors [updated v1020012; cited 2014].

[28] Smith TJ, Khatcheressian J, Lyman GH, Ozer H, Armitage JO, Balducci L, et al 2006 update of recommendations for the use of white blood cell growth factors: an evidence-based clinical practice guideline. Journal of clinical oncology: official journal of the American Society of Clinical Oncology. 2006;24(19):3187–205. Epub 2006/05/10. doi: 10.1200/jco.2006.06.4451 . 16682719 10.1200/JCO.2006.06.4451

[29] Ethier M-C, Blanco E, Lehrnbecher T, Sung L. Lack of clarity in the definition of treatment-related mortality: pediatric acute leukemia and adult acute promyelocytic leukemia as examples2011 2011-11-10 00:00:00. 5080–3 p. 10.1182/blood-2011-07-363333 21937689

[30] Bates I, Chapotera GK, McKew S, van den Broek N. Maternal mortality in sub-Saharan Africa: the contribution of ineffective blood transfusion services. BJOG: an international journal of obstetrics and gynaecology. 2008;115(11):1331–9. Epub 2008/10/01. doi: 10.1111/j.1471-0528.2008.01866.x . 18823485 10.1111/j.1471-0528.2008.01866.x

[31] Field SP, Allain J-P. Transfusion in sub-Saharan Africa: does a Western model fit? Journal of Clinical Pathology. 2007;60(10):1073–5. doi: 10.1136/jcp.2006.043505 17412874 10.1136/jcp.2006.043505 PMC2014840

[32] Orem J, Sandin S, Weibull CE, Odida M, Wabinga H, Mbidde E, et al Agreement between diagnoses of childhood lymphoma assigned in Uganda and by an international reference laboratory. Clinical epidemiology. 2012;4:339–47. Epub 2013/01/02. doi: 10.2147/CLEP.S35671 ; PubMed Central PMCID: PMCPMC3531988. 23277743 10.2147/CLEP.S35671 PMC3531988

